# Adaptive Triboelectric Nanogenerators for Long-Term Self-Treatment: A Review

**DOI:** 10.3390/bios12121127

**Published:** 2022-12-05

**Authors:** Zequan Zhao, Yin Lu, Yajun Mi, Jiajing Meng, Xueqing Wang, Xia Cao, Ning Wang

**Affiliations:** 1 Center for Green Innovation, School of Mathematics and Physics, University of Science and Technology Beijing, Beijing 100083, China; 2 Beijing Institute of Nanoenergy and Nanosystems, Chinese Academy of Sciences, Beijing 100083, China; 3 School of Chemistry and Biological Engineering, University of Science and Technology Beijing, Beijing 100083, China

**Keywords:** triboelectric nanogenerator, self-powered device, adaptivity, clinical treatment, self-diagnosis

## Abstract

Triboelectric nanogenerators (TENGs) were initially invented as an innovative energy−harvesting technology for scavenging mechanical energy from our bodies or the ambient environment. Through adaptive customization design, TENGs have also become a promising player in the self-powered wearable medical market for improving physical fitness and sustaining a healthy lifestyle. In addition to simultaneously harvesting our body’s mechanical energy and actively detecting our physiological parameters and metabolic status, TENGs can also provide personalized medical treatment solutions in a self-powered modality. This review aims to cover the recent advances in TENG-based electronics in clinical applications, beginning from the basic working principles of TENGs and their general operation modes, continuing to the harvesting of bioenergy from the human body, and arriving at their adaptive design toward applications in chronic disease diagnosis and long-term clinical treatment. Considering the highly personalized usage scenarios, special attention is paid to customized modules that are based on TENGs and support complex medical treatments, where sustainability, biodegradability, compliance, and bio-friendliness may be critical for the operation of clinical systems. While this review provides a comprehensive understanding of TENG-based clinical devices that aims to reach a high level of technological readiness, the challenges and shortcomings of TENG-based clinical devices are also highlighted, with the expectation of providing a useful reference for the further development of such customized healthcare systems and the transfer of their technical capabilities into real-life patient care.

## 1. Introduction

Since their initial invention in 2012, the triboelectric nanogenerators (TENGs) have attracted extensive attention due to their tremendous potential in the fields of energy harvesting and active sensing. Currently, TENGs are undergoing rapid progress in a variety of other fields, such as green energy, molecular detection, health care, and gesture recognition, due to characteristics that make them exceptional for application in self-powered electronic systems, where TENGs can act as both power sources and as smart sensors, including as dynamic force sensors and chemical sensors, with intrinsic sensitivity toward chemical reactions at the triboelectric interface [1,2]. As a result, TENGs could possibly become a part of people’s daily lives in the form of either power accessories or active sensors, or, in some cases, both [3].

In the field of health care and clinical treatment, bio−friendly TENGs can be worn directly on or implanted into the body, and thus can be used to observe and analyze the patient’s physiological parameters and metabolic status, and can even be used to treat diseases with the support of various information technologies, such as wireless connection, cloud services, and information storage [4]. With advantages that include simple operation and easy miniaturization, TENGs can be adaptively designed and seamlessly embedded into intelligent systems for the scavenging of mechanical energy from either the ambient environment or the human body, thus endowing the system with features such as sustainability, wearability, and portability. As a result, the application of TENGs in the field of clinical treatment would help to meet the requirement for clinical devices with predictive, personalized, and participatory characteristics, making it possible to simultaneously detect physiological signals, maintain sustainable operation, and provide continuous exercise guidance while harvesting bioenergy, thus achieving in vivo, accurate and intelligent self-treatment and self-monitoring [5,6].

In addition to enabling the measurement of clinically relevant parameters and showing the health status of individuals [7,8,9,10,11,12,13,14,15,16], TENGs can complement traditional power sources, which are generally fabricated using toxic chemicals and have limited capacity. The introduction of TENGs into the operation and implementation of the above-mentioned systems could alleviate the dependence on power sources and sensor technology, thus increasing wireless mobility, interactivity, and intelligence [17,18,19,20,21,22]. TENGs can be especially helpful in the treatment of chronic stubborn disorders, in which rapid recovery often fails to be achieved. In these cases, long-term follow-up is the most effective approach for improving prognosis, including long-term collection and analysis of body data, along with continuous personalized treatment. Depending on the patient’s disease type and needs, treatment may unavoidably involve flexible and highly customized biomedical equipment requiring sustainable operation [23].

**Figure 1:**
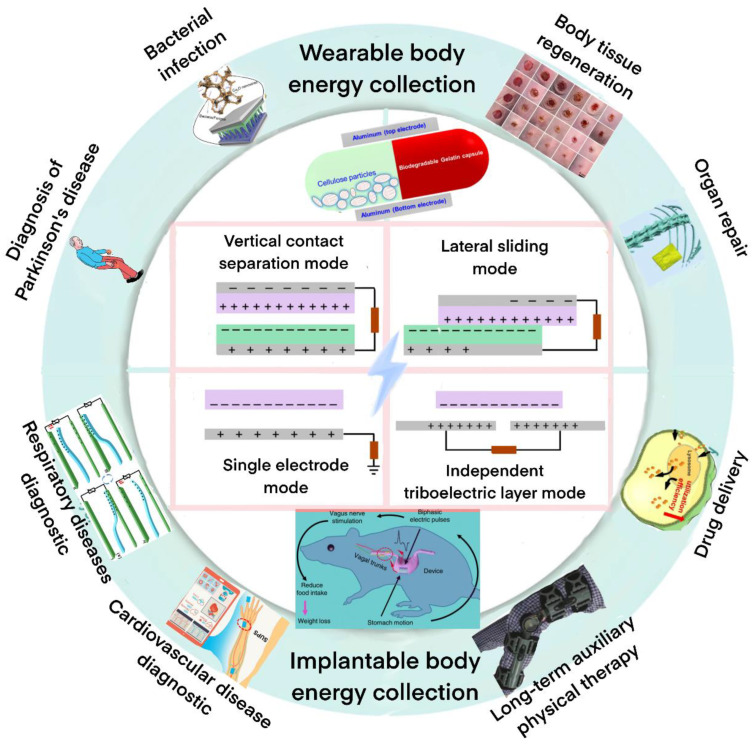
Triboelectric nanogenerators for long-term self-treatment and self-diagnosis.

Therefore, this review aims to cover the advances in TENGs and TENG-based wearable sensor technology (Figure 1), which present enormous opportunities for their deployment in health care [24,25,26,27], especially in connected health care and long-term personalized treatment, in the context of which TENGs can provide both high-quality, real-time measurement of personal health parameters as well as a long-lasting power source [28]. In addition to discussing the current application of TENG-based wearable devices in health care, including daily health and safety monitoring and the use of TENGs in clinical practice and treatment, we also emphasize their current shortcomings and suggest directions for further research.

## 2. Biological Energy Collection

Considering their personalized usage scenarios, TENG-based devices will be able to support more complex functional modules in order to meet the needs of different groups of users, requiring TENGs with higher power output. The key to solving this problem is to expand the methods of biological energy collection, thus improving efficiency [29,30,31,32,33].

### 2.1. Working Principle of TENG

**Figure 2:**
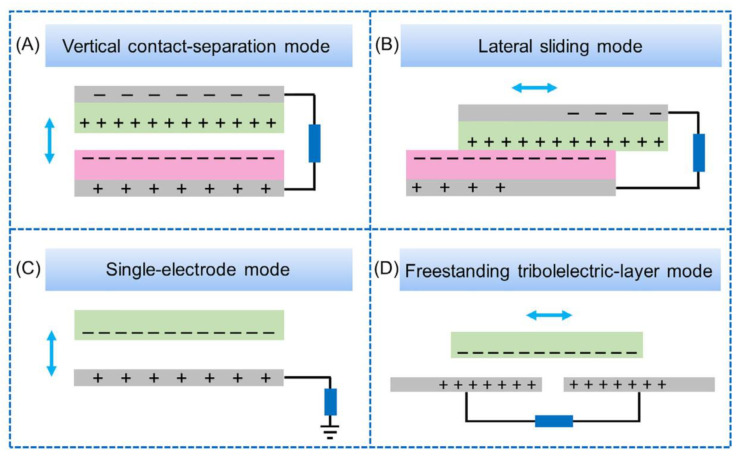
(**a**) Vertical contact-separation mode. (**b**) Lateral sliding mode. (**c**) Single-electrode mode. (**d**) Freestanding triboelectric-layer mode. Reprinted with permission from [39]. 2020, copyright John Wiley and Sons.

TENGs mainly use the triboelectric effect and electrostatic induction coupling to convert biomechanical energy into electrical energy to power medical equipment. Theoretically, when two materials with different degrees of electronegativity come into contact, electrons will flow between them. When they are separated, due to the electrostatic induction effect, electrons will flow to the external load. Upon repetition of the above process, the TENG will output alternating current [34]. On this basis, TENGs can be divided into four working modes (Figure 2): vertical contact-separation mode [35], lateral sliding mode [36], single-electrode mode [37], and independent triboelectric layer mode [38].

Vertical contact-separation (CS) mode: When two objects with different degrees of electronegativity are in vertical contact, electrons are exchanged at the contact surface. Then, when two objects are separated, due to there being an equal number of opposite charges between them, electrostatic induction leads to a potential difference between the electrodes attached to them, generating electric current when connecting through the wires, and then the potential difference gradually disappears. When the two separated objects come into contact again, the opposite potential difference is generated between the two electrodes again, thus generating opposite currents through the wires. With the repetition of contact-separation cycles, alternating current is output.

Lateral sliding (LS) mode: The principle of lateral sliding is similar to that of the vertical Contact-separation mode, except that the relative displacement is changed from vertical to horizontal. Alternating current is output as a result of repeated displacement in the horizontal direction.

Single-electrode (SE) mode: This is the simplest mode in terms of structure. The method takes the earth as an electrode and generates a potential difference between the metal electrode and the earth through electrostatic induction, thus producing current.

Independent triboelectric layer (FT) mode: FT mode involves placing the charged object between two electrodes, which are attached to the dielectric layer. When the charged object moves between the two electrodes, the potential difference between the electrodes changes, thus generating current.

Theoretically, due to the low requirements of TENG power generation, a variety of friction layer materials and electrode materials are available to choose from. The TENG can then be designed according to the personalized needs of patients.

### 2.2. Wearable Body Energy Collection

**Figure 3:**
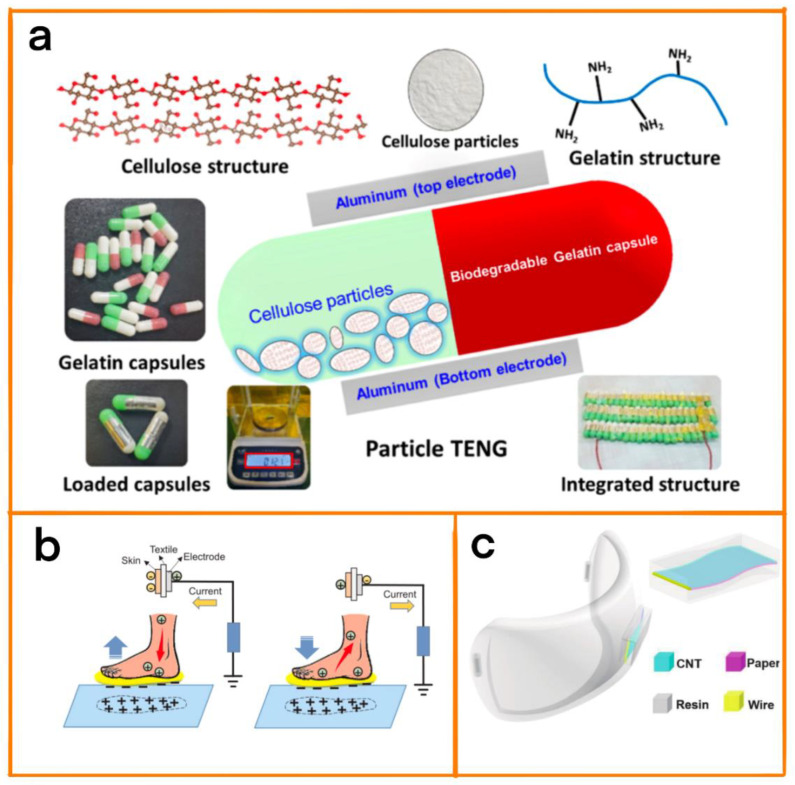
(**a**) Design scheme and schematic diagram of the rapid degradation of P-TENGs showing the tiny cellulose particles and gelatin capsules. Reprinted with permission from [41]. 2022, Elsevier. (**b**) Schematic diagram of the working mechanism of the single-electrode mode triboelectric system. Reprinted with permission from [42]. 2019, Elsevier. (**c**) Structure diagram of the 3D-printed breath-driven TENG. Reprinted with permission from [43]. 2022, Springer Nature.

TENGs can be customized to a certain extent according to the energy collection requirements due to the possibility of selecting diverse raw materials and their loose structural design requirements. Therefore, appropriate materials and shapes can be selected for the production of TENGs through the process of adaptive design in order to be able to collect the bioenergy dispersed in the human body and to maintain a certain degree of biological friendliness [40]. Saqib et al. proposed a TENG that was able to obtain energy from omnidirectional movements in the human body (Figure 3a [41]) The TENG was made by placing cellulose-based particles in rapidly degradable gelatin capsules. Small cellulose particles (~6 µ m) were used as friction anode materials, and gelatin capsules were used as friction cathode layers. When tested, the power harvested by the TENG ranged from 5.488 to 70 μW, with a maximum energy conversion efficiency of 74.35%. Due to the advantages of all-around energy collection, the TENG had a wider energy collection channel, which was able to perform energy collection under complex motion while maintaining a high energy collection efficiency.

Park and others ingeniously used the electric charge generated by friction in the human body to make simple aluminum electrode triboelectric nanogenerators (Figure 3b) [42]. The system collects the frictional electric energy between the sole and the ground through the electrostatic induction of aluminum electrodes.In addition, the power generated in each step is enough to instantly light 100 commercial light-emitting diodes (LEDs). Although this aluminum electrode TENG has the advantages of easy fabrication, its simple structure also leads to it having unsatisfactory energy conversion efficiency. Zhu et al. integrated TENGs into a mask via 3D printing, and produced breath-driven TENGs (Figure 3c) [43]. The TENG generates electrical signals corresponding to the breath airflow and extracts personal information during breathing. This 3D-printed TENG has the advantage of it being easy to perform shape customization and meet the personalized needs of different people.

Wang’s team developed a new TENG using conductive elastic sponges (ES-TENG) [44]. This soft and special structure has great flexibility, and the adaptive deformation of the sponge enables it to collect kinetic energy from tumbling motions of different amplitudes from different surfaces of flexible objects, thus effectively improving the efficiency of energy collection. In addition, as a result of its adaptive design, this TENG can be easily installed on the body in order to be able to collect energy from multi-angle body movement.

Compared with traditional power generation methods, the collection of human motion energy using wearable devices avoids bloated power design and has the advantages of small size, flexibility, stability, etc.

### 2.3. Implantable Body Energy Collection

**Figure 4:**
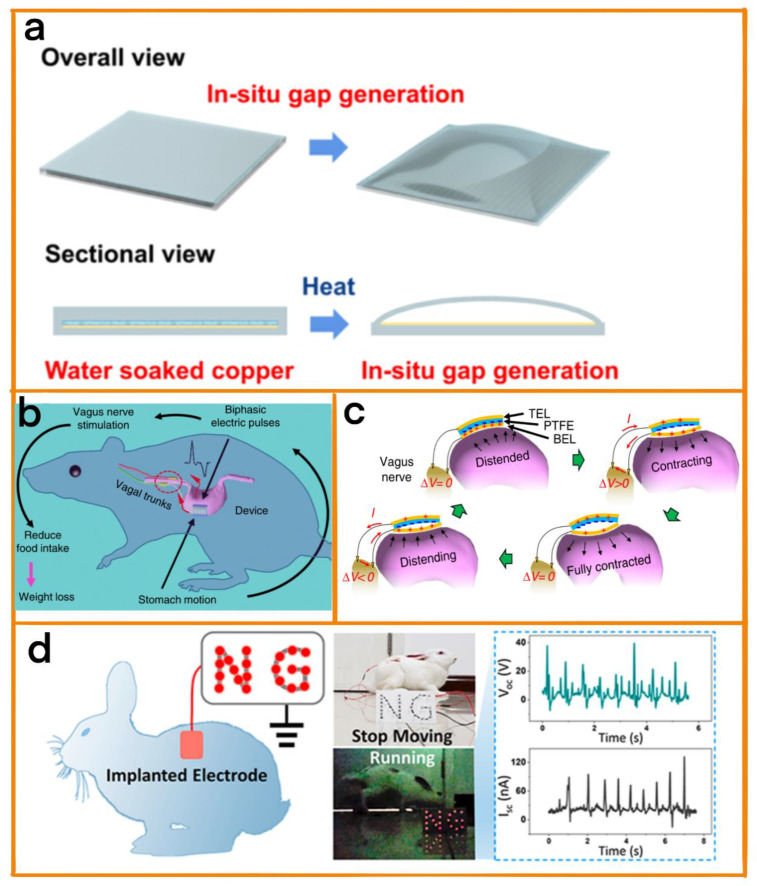
(**a**) Schematic diagram briefly showing the in situ air gap generation of NSTENG. Reprinted with permission from [49]. 2021, Elsevier. (**b**) Working principle of the relevant vagus nerve stimulation system, schematically showing the generation of two-phase electrical signals and the path of vagus nerve stimulation. (**c**) Schematic diagram of the power generation principle of the vagus nerve stimulation device at different gastric motility stages. Reprinted with permission from [50]. 2018, nature. (**d**) LED bulbs lit by BISS implanted in rabbits and the open circuit voltage and short circuit current of BISS during operation. Reprinted with permission from [52]. 2019, ACS.

For implantable medical devices, there is a risk of exposure due to wounds and rejection by the human immune system. This makes it necessary to control the number of operations and the biological safety of medical equipment. The use of TENGs for the collection of biological energy from the human body can guarantee the long-term operation of the implanted medical device, thereby reducing the number of surgeries required to change batteries. In addition, the extensive selection of raw materials usable for TENGs provides multiple channels for biological safety [45,46,47,48]. Zhao et al. developed a TENG [49] that could be used to obtain energy from the beating of the heart. The TENG was used to generate a new environmentally friendly in situ gap through the evaporation of distilled water in which it was soaked (Figure 4a), which was then used to manufacture a no-spacer triboelectric nanogenerator (NSTENG). This unique manufacturing method ensures biological safety and avoids pollution in the body. In addition, when installed on a rat heart, it was also able to monitor normal heart movement, with the accuracy of the heart rate measurements reaching as high as 99.73%. Implantable TENGs provide the possibility of developing self-powered heart detection instrument in the future.

Yao et al. introduced a vagus nerve stimulation system that generates electricity through gastric peristalsis (Figure 4c) [50]. The system includes a flexible, biocompatible nanogenerator attached to the surface of the stomach. This generates biphasic electrical pulses in response to gastric peristalsis, which can also be used to control the weight of rats by stimulating the vagus nerve to reduce food intake (Figure 4b).

In 2018, Liu et al. proposed a new endocardial pressure monitoring technology based on implantable TENG, which used inductively coupled plasma and corona discharge to conduct triboelectric treatment on polytetrafluoroethylene film, so that its output increased to 6.2 V. To improve biological safety, flexible PDMS with biocompatibility and blood compatibility was selected for the packaging of the device, with a size of 1.0 cm × 1.5 cm × 0.1 cm. In addition, the device could also be used to monitor cardiovascular diseases such as ventricular premature beat and ventricular fibrillation by detecting the ventricular EP of pigs [51].

Shi et al. proposed a TENG that was able to generate electricity through subcutaneous muscle movement (M-TENG) [52]. The system has a straightforward structure, including only one electrode. Immersing it in the body makes it possible to convert biological activity into electrical energy. For example, a 1.5 cm × 2 cm titanium alloy film was implanted between the skin and muscle layers on the back of the rabbit, and the titanium alloy film was connected to the LED bulb through wires. Then, the rhythmic flashing of the LED bulb could be observed when the rabbit moved (Figure 4d). Different power generation methods and raw materials affect the output of TENG. We can compare different TENG outputs to find out the suitable device (Table 1).

## 3. Real-Time Medical Diagnostic Equipment

The vast majority of diseases cause a gradual deterioration in the patient over time, and the rapid detection and treatment of diseases in their early stages can not only greatly reduce the investment in medical treatment required for patients, but also effectively reduce the severity of sequelae. TENG-based real-time medical monitoring equipment can provide a variety of physiological data measurements of the human body over a long time. And the equipment has different outputs with different raw materials and testing positions (Table 2). Through terminal data analysis and processing, diseases can be quickly diagnosed, and personalized treatment plans can be provided for patients, effectively improving the use of medical resources.

### 3.1. Diagnostic Equipment for Cardiovascular Disease 

As a disease of the circulatory system, cardiovascular disease includes heart disease and vascular disease. The main diagnostic methods for heart disease include invasive and non−invasive examinations [53]. Invasive tests, including cardiovascular endoscopy, endomyocardial biopsy, and other studies, which may cause trauma to patients, come with certain risks. Noninvasive investigations, including ECG and echocardiography, are not traumatic, but have low diagnostic value and diagnosis cannot be performed in real time over a long time. Therefore, finding new diagnosis methods is key to solving these problems.

#### 3.1.1. Blood Pressure Diagnosis

**Figure 5:**
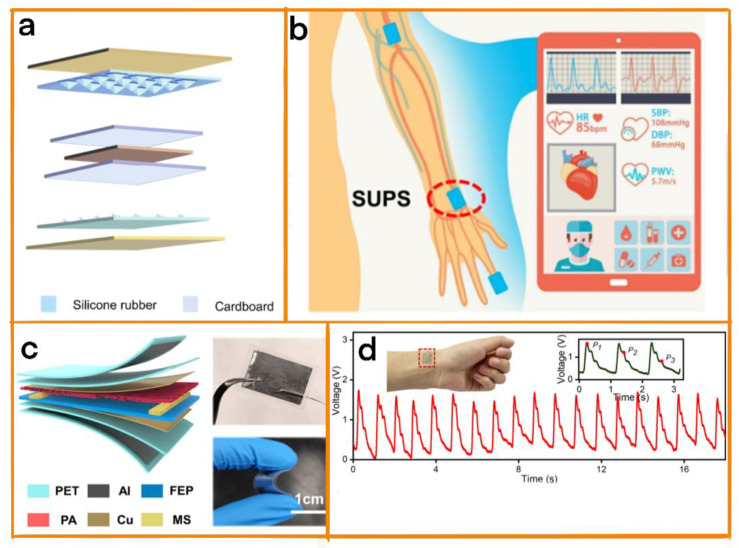
(**a**) Triboelectric nanogenerator sensor with a double sandwich structure. Reprinted with permission from [54]. 2022, Nano Research. (**b**) Integrated description of SUPS for non-invasive multi-index pulse monitoring. (**c**) Schematic diagram of the structure of SUPS. Optical photo of the built SUPS. Reprinted with permission from [55]. 2021, Elsevier. (**d**) FPS test radial pulse wave. Reprinted with permission from [56]. 2022, Elsevier.

Blood pressure detection is a key means of diagnosing cardiovascular diseases. Effective long-term collection blood pressure data could help to analyze personal health changes, thus assisting in determining the causes of diseases. Doctors are able to analyze patients’ comprehensive physical conditions on the basis of long-term data, and provide constructive suggestions with the aim of providing patients with personalized medical care. TENG-based blood pressure monitoring instruments are not only characterized by the use of lightweight and safe raw materials, they are also able to guarantee the long-term stable operation of the instrument because of its self-powered characteristics. Ran et al. developed a cuff-free, self-powered continuous blood pressure monitoring system. The system was based on a new double sandwich structure (a copper electrode sandwiched between two layers of silicone rubber and cardboard is added at the outermost layer to provide support) (Figure 5a) [54]. Using the adaptive design described above, this system was able to achieve a sensitivity of 0.89 V/kPa, and a response time of 32 ms; this sensor was easily able to capture blood vessel signals, and could be used for long-term and efficient real−time blood pressure diagnosis.

#### 3.1.2. Pulse Diagnosis

Real−time pulse diagnosis can be used to provide doctors with continuous information on the pulse rhythm information of patients. On the basis of the analysis of this information, clues can be found as early as possible of certain cardiovascular diseases, such as atrial fibrillation, supraventricular tachycardia, premature beats, etc., and they can be treated in a timely fashion. At the same time, long-term pulse information can indicate direction for doctors to follow in analyzing the heart rhythms of patients, thus providing strong support for the further personalization of the treatment of patients. Xu et al. designed a self-powered sensitive ultra-pulse sensor (SUPS) that could be used to conduct long-term non-invasive real-time cardiovascular monitoring (Figure 5c) [55]. The device uses an FEP film consisting of a nanowire array and a Polyamide (PA) film with a fiber structure as the triboelectric layer, copper foil as the electrode, with the addition of a melamine sponge with a porous structure as an interlayer between the triboelectric layers. As a result of the adaptive design described above, SUPS showed excellent sensing performance, including a super sensitivity of 10.29 nA/kPa, a low detection limit of 5 mg, and a rapid response time of 30 ms. It can be used to monitor cardiovascular systems in a long-term, stable and accurate manner, and is expected to be used in the diagnosis and prevention of cardiovascular diseases (Figure 5b).

Wang and his team proposed a flexible pressure sensor (FPS) for measuring the pulse of the cutaneous artery (Figure 5d) [56]. PTFE, copper powder, and conductive double-sided adhesive were used to perform detection following the TENG principle, and this was demonstrated to be suitable for the diagnosis of cardiovascular disease. This device was produced by means of a convenient coating operation, by which a copper powder layer with a natural microstructure with a 500-nanometer scale was formed on a conductive double-sided adhesive. This hierarchical microstructure was composed of copper powder, making the FPS sensitive to weak pressure signals; the FPS had a high sensitivity of 1.65 V/kPa, and was able to accurately detect arteries over a long period of time, thus making it useful for the diagnosis of cardiovascular diseases.

### 3.2. Diagnostic Equipment for Respiratory Diseases 

**Figure 6:**
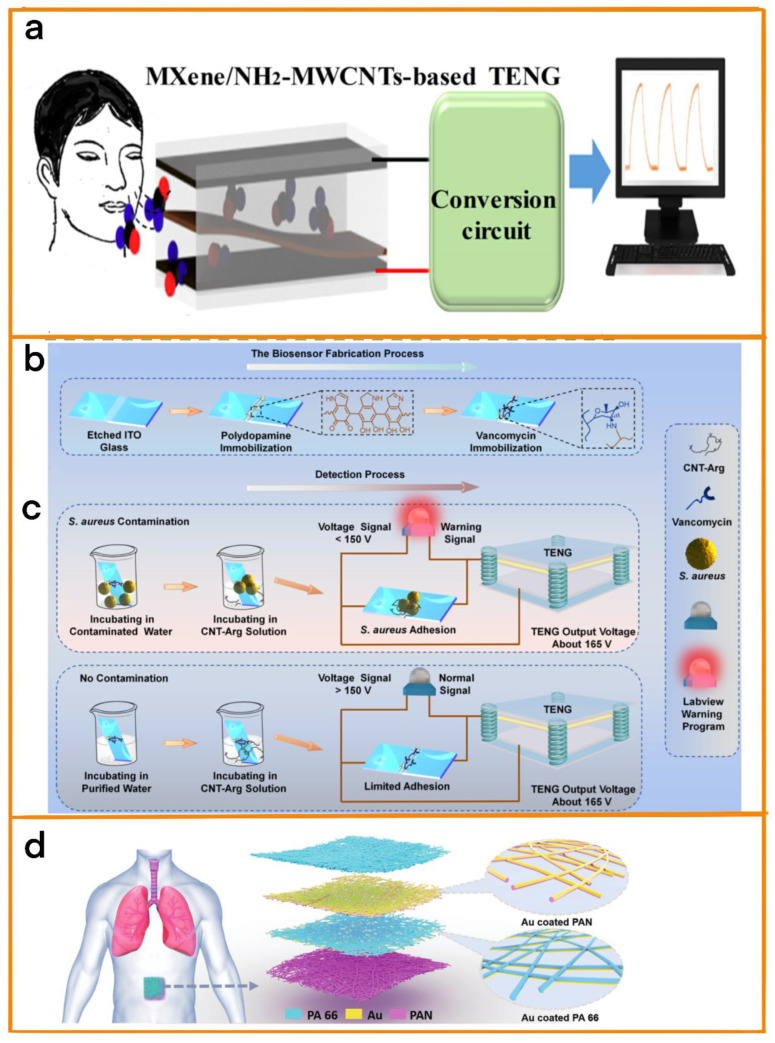
(**a**) Application diagram of MXene/NH2−MWCNTs-based TENG. Reprinted with permission from [60]. 2022, Elsevier. (**b**) The preparation process of ITO-Van, which is able to specifically capture Staphylococcus aureus, including the immobilization of dopamine and vancomycin on the etched surface of ITO glass. (**c**) Schematic diagram of staphylococcus aureus detection using a self-powered biosensor system. In the process of detecting Staphylococcus aureus, a liquid environment is assumed, and a vertical contact-separation TENG is used as the voltage signal source. Reprinted with permission from [62]. 2022, Elsevier. (**d**) SANES attached to the abdominal surface used in a respiratory monitoring application scenario; SANES schematic diagram; and an enlarged view of PAN nanofiber film and PA 66 nanofiber film coated with an Au electrode layer. Reprinted with permission from [63]. 2021, Elsevier.

Presently, a variety of methods can be employed for the diagnosis of respiratory diseases, including imaging diagnosis and endoscopy [57,58,59]. However, these conventional detection methods are not able to transmit the progress of disease in real time in cases of long-term diseases such as chronic rhinitis and pharyngitis. This leads to doctors being unable to quickly change treatment strategies, which affects the efficiency of treatment. In addition, the costly and time-consuming nature of testing equipment also affects patients’ quality of life to varying degrees. New breath detection devices based on TENGs could find easy application for long-term operation due to their being self-powered and stable. In addition, because of their small size, light weight, and other advantages, they can be customized to a certain extent depending on the individual needs of patients, thus minimizing their impact on the lives of patients. For example, Zhang and his team proposed a TENG for use in the self−powered detection of exhaled gas and disease diagnosis (Figure 6a) [60]. The TENG was made of Ti_3_C_2_Tx MXene/amino functionalized multi-wall carbon nanotubes (MXene/NH_2_-MWCNTs). It was driven by respiration, and could be used to determine the type of respiration on the basis of voltage changes in order to diagnose respiratory disease. Furthermore, on the basis of the long-term respiratory data collected, the physical condition of the patient and disease progression can be analyzed in detail. Additionally, since MXene/NH_2_-MWCNTs are sensitive to formaldehyde gas, the device is also able to accurately detect formaldehyde gas in exhaled gas, which could play an important role in the determination of air safety.

#### 3.2.1. Diagnosis of Diseases Caused by Infection with Gram-Positive Bacteria

Gram−positive bacteria such as Staphylococcus aureus can cause a series of diseases, such as upper respiratory tract infection, suppurative tonsillitis, bronchitis, pneumonia, and skin and surgical incision infections. They pose a significant challenge to human medical treatment. In addition, most bacterial infections cause a deterioration in the patient over time. Early detection and treatment can reduce wastage of medical resources and effectively reduce the severity of sequelae. Considering that routine invasive testing increases the risk of multiple infections in patients, it is urgently necessary to find non−invasive and efficient detection methods, including surface-enhanced Raman spectroscopy (SERS) and highly sensitive detection methods using TENGs.

Ma and his team, using Au@Ag NPs/slide as an enhanced substrate, constructed an aptamer-based SERS method for detecting Staphylococcus aureus. The ROX-aptamer of *S. aureus* was modified on the surface of Au@Ag NPs/slide by means of electrostatic interaction [61]. Because the aptamer is able to specifically bind to Staphylococcus aureus, it will cause the rox-aptamer to fall off the substrate surface, thus reducing the SERS signal intensity of the substrate. Then, the target bacteria can be successfully detected by analyzing the signal changes of SERS.

In this regard, Wang et al. developed a TENG especially for detecting Gram-positive bacteria in solution in order to be able to diagnose relevant diseases in time [62]. The system immobilized polyamine and vancomycin on the etched surface of ITO glass and recognized Gram-positive bacteria by means of vancomycin bacterial wall interaction (Figure 6b). Guanidine-functionalized multi-wall carbon nanotubes (CNT Arg) were used as signal amplification materials. Then, the system was able to specifically detect Gram-positive bacteria in solution by measuring the voltage change in the biosensor (Figure 6c). This equipment could be used to observe Gram-positive bacteria stably over a long time, and can be applied for the diagnosis of diseases resulting from infection with Gram-positive bacteria or for the determination of water quality in the future.

**Table 2:** Summary of TENG parameters.

Date	Positions	Sizes [cm^2^]	Materials	Energy Sources	Outputs	Applications	Working Modes
2022 [54]	Pulse	5 × 5	Cu, Silicone rubber	Pulse vibration	0.89 V/kPa	Pulse monitoring	Contact-separation
2021 [55]	Pulse	None	FEP, PA, Cu, PET	Pulse vibration	10.29 nA/kPa	Pulse monitoring	Contact-separation
2022 [56]	Pulse	1.8 × 1.6	PTFE, Cu	Pulse vibration	1.65 V/kPa	Pulse monitoring	Contact-separation
2022 [60]	Nose	5 × 2	MXene	Respiratory drive	27 μW	Respiratory monitoring	Contact-separation
2022 [62]	Solution	2 × 0.5	ITO	Vibration	165 V	Bacterial detection	Contact-separation
2021 [63]	Nose	4 × 4	Polyacrylonitrile, Polyamide 66	Respiratory drive	420 v	OSAHS diagnostics	Contact-separation
2021 [64]	Wearable	31 × 3	Catechol, Chitosan, Diatom	Movement	29.8 mW/m^2^	Parkinson diagnosis	Contact-separation
2021 [65]	Wearable	4 × 3.5	Ecoflex, Al	Movement	2 V	Parkinson diagnosis	Contact-separation

#### 3.2.2. Diagnosis of Obstructive Sleep Apnea-Hypopnea Syndrome

Obstructive sleep apnea-hypopnea syndrome (OSAHS) affects about 1 billion people worldwide. This disease can cause repeated upper airway collapse during sleep, further leading to intermittent hypoxia, sleep disorders, and even cerebrovascular diseases. At the same time, most OSAHS remains diagnosed. To solve this problem, Peng et al. prepared a respirable electronic skin (SANES) based on TENGs for real-time respiratory monitoring and diagnosis of OSAHS [63]. It was prepared using gold as an electrode, multilayer polyacrylonitrile, and “polyamide 66” nanofiber as positive and negative friction electrodes (Figure 6d); the highest pressure sensitivity obtained was 0.217 kPa^−1^, and it also showed good air permeability. Therefore, the electronic skin was able to achieve accurate breath detection. At the same time, the team further developed a self−powered diagnostic system for the assessment of OSAHS severity. The automatic diagnostic system was able to diagnose OSAHS in real time, effectively prevent the occurrence of OSAHS, and improve sleep quality.

### 3.3. Diagnosis of Parkinson’s Disease

**Figure 7:**
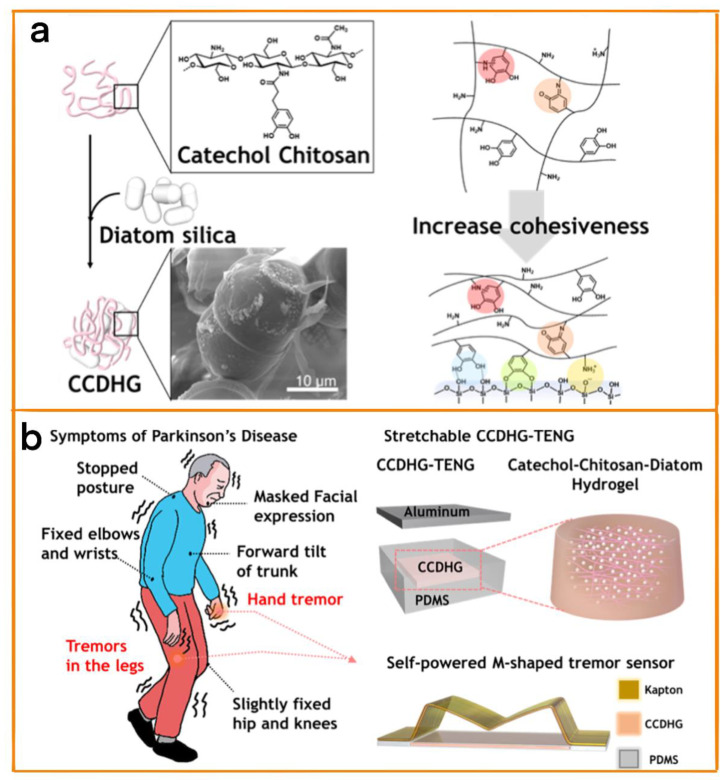
(**a**) Preparation method of catechol chitosan diatom hydrogel, and the mechanism of enhancing the cohesion of catechol chitosan diatom hydrogel. Optical image. (**b**) Typical symptoms of Parkinson’s disease. Schematic diagram of catechol chitosan diatom hydrogel triboelectric nanogenerator and vibration sensor. Reprinted with permission from [64] 2021, Elsevier.

Parkinson’s disease is a common degenerative disease of the elderly nervous system that leads to motor disorders in patients. At present, doctors mainly rely on careful observation to assess the development of the disease. Therefore, Kim et al. synthesized a highly stretchable and self-healing hydrogel TENG with natural biomaterials (catechol chitosan was mixed with pill-shaped diatom frustules with sizes of 20–50 µm) [64] (Figure 7a) that had an instantaneous power density of 29.8 mW/m^2^. The TENG obtains energy from human motion and combines it with the M-type Kapton film to form a self-powered tremor sensor. The sensor was able to diagnose and monitor Parkinson’s disease through the measurement of the low-frequency motion of patients in combination with machine learning algorithms (Figure 7b). The individual needs of different patients can be met by changing the appearance structure. At the same time, Yuce et al. developed a self-powered TENG for diagnosis of Parkinson’s disease [65]. This nanogenerator was made of flexible materials, and through customization was able to meet the need for patients to be able to wear it for a long time. It consisted of a 4 cm × 3.5 cm dielectric and 2 mm × 3 mm aluminum electrodes. When the patient’s hand bends, the system generates voltage due to the change in the relative position of the dielectric material and the aluminum electrode. This sensor can be used to evaluate the patient’s condition, and can be worn for long-term monitoring due to its self-powering characteristics.

## 4. Long-Term Self-Treatment Equipment

Compared with acute diseases that can be effectively treated in a short time, rapid recovery often cannot be achieved for stubborn chronic diseases. In these cases, long-term customized treatment in accordance with the needs of patients is an effective way of solving this problem, but this inevitably involves the use of flexible and highly customized biomedical equipment. Biomedical equipment based on TENGs has a comparative advantage in terms of customization due to its self-powered character and flexible appearance. At the same time, the wide selection of raw materials from which TENGs can be produced also give them more room for development in terms of biodegradability, compliance, biological friendliness, and different energy collection methods and outputs (Table 3).

### 4.1. Body Tissue Regeneration

Self-powered flexible equipment can find wide application in various fields, and multifunctionality is always highly appreciated because it provides the advantages of miniaturization, higher integration, and lower power consumption.

#### 4.1.1. Nerve Tissue Regeneration

**Figure 8:**
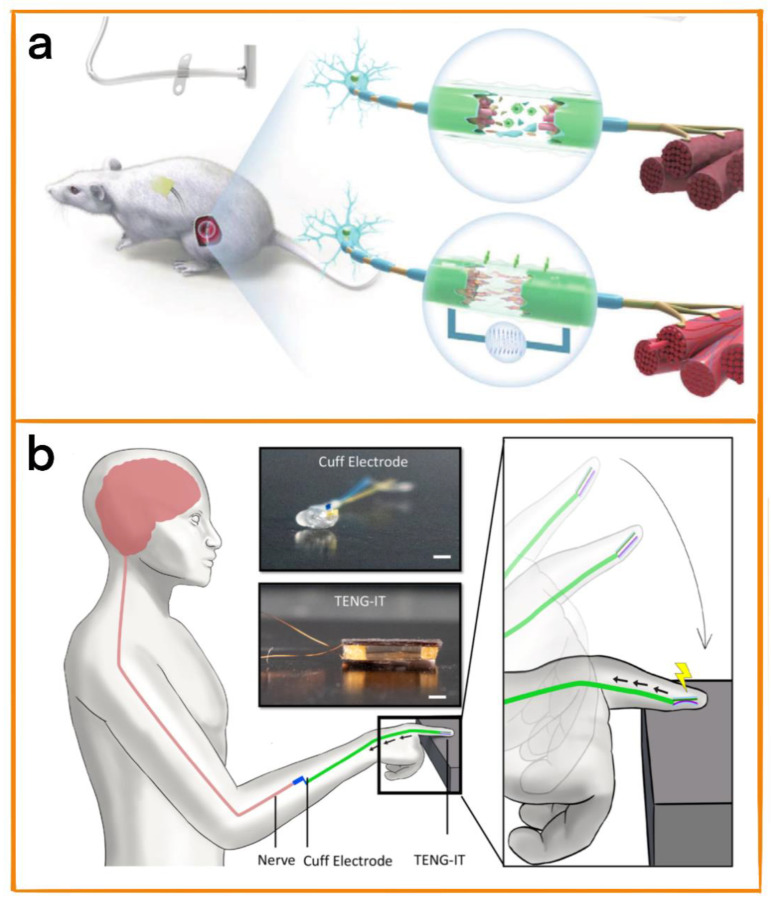
(**a**) Schematic diagram of electrode implant solution. Cut the skin, and introduce an electric stimulation cuff on the left sciatic nerve of the rats. Place Cs TENG as an energy collector at the waist of the rat, and connect the electrode with the encapsulated platinum wire to ensure that the damaged area is located between the two electrodes. Reprinted with permission from [69]. 2022, John Wiley and Sons. (**b**) Use of TENG-IT to restore touch. TENG-IT is implanted under the skin (desensitized fingers). Reprinted with permission from [70]. 2021, ACS.

Peripheral nerve injury is one of the common causes of disability, and it often requires a long duration of regular treatment to recover, even requiring treatment via serious nerve anastomosis surgery. Nerve stimulation devices based on TENGs can provide long-term and stable electrical stimulation, effectively promoting nerve regeneration and increasing the efficiency of treatment in combination with traditional treatment methods, and the high degree of possible customization of TENGs means that they can be adapted to the different injury conditions of different patients, thus reducing the discomfort of patients during the healing process [66,67,68]. Therefore, Zhou et al. developed an implanted sciatic nerve stimulation system (ISR-NES) that was able to effectively promote the regeneration of the sciatic nerve [69]. The system’s Contact-separation triboelectric nanogenerator (Cs TENG) spontaneously generated a biphasic electric pulse in response to respiratory and body movement. Then, the electric pulse stimulated the injured sciatic nerve through the cuff electrode in order to repair it (Figure 8a). PDMS and polyamide 6 (PA6) films were selected as the friction layer of the TENG, and aluminum foil was pasted on the back of the two friction layers as the conductive layer. Because the vibration amplitude generated by the breathing of rats is small, it is difficult to collect energy using ordinary TENGs; therefore, the sensitivity of the TENG was improved using the spacing structure through adaptive design: the dielectric films of the two friction layers were stacked face to face, a surface charge with the opposite sign forms on the two contact surfaces, an air gap is formed in the middle during separation, and an induced potential difference is formed between the two electrodes. By menas of the above design, the output of the TENG was successfully improved. Due to the flexibility of the raw materials used, the system demonstrated high customizability and a stable power supply. In the future, it will be able to meet the personalized needs of patients and provide them with targeted long-term treatment.

Tactile loss is one of the more common outcomes of peripheral nerve injury. Implantable nerve prostheses represent a promising direction, but these still possess some disadvantages, including the complexity of their use scenarios and the requirement of bloated external power supplies. Therefore, to solve this problem, Shlomy et al. proposed triboelectric nanogenerators (TENG-IT) with simple structures that were self-powered, biocompatible, and highly customizable for tactile restoration [70]. PDMS, nylon (Ny), and cellulose acetate butyrate (CAB), which has the advantages of flexibility and biocompatibility, were selected as friction layers in this device, with PDMS being a negative layer, and Ny and CAB being positive layers. The device was implanted under human skin in order to convert pressure into potential, which was transmitted to healthy sensory nerves through cuff electrodes (Figure 8b). As a therapeutic device intended for long-term implantation, the personalized design enabled by its simple structure can result in the improvement of a patient’s quality of life to a certain extent.

#### 4.1.2. Connective Tissue Regeneration

**Figure 9:**
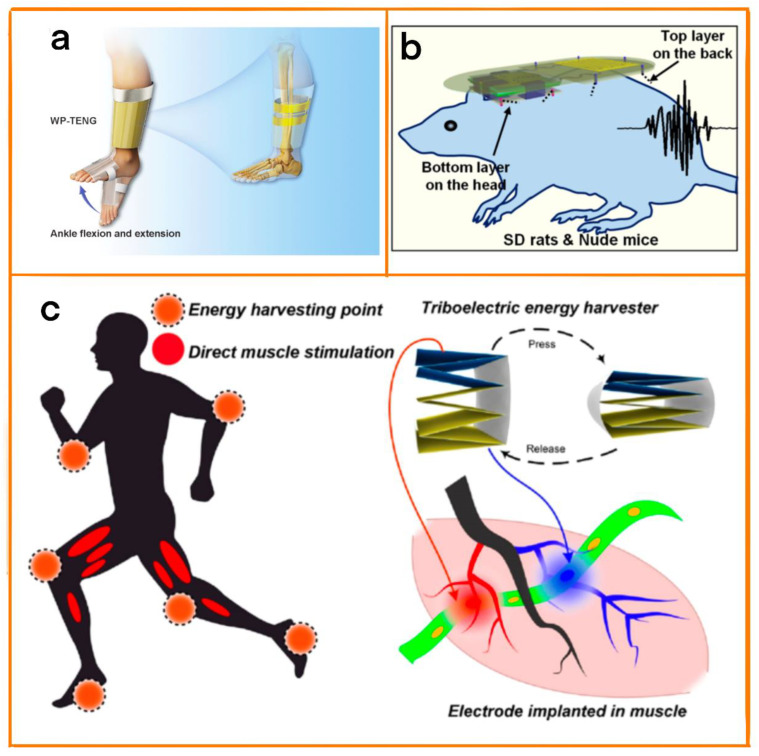
(**a**) Human limb movement activates the WP−TENG to achieve bone repair. Reprinted with permission from [71]. 2022, John Wiley and Sons. (**b**) Schematic diagram of M-ESD system for hair regeneration. Reprinted with permission from [74]. 2019, ACS. (**c**) Electrical muscle stimulation powered by TENGs. Reprinted with permission from [77]. 2019, ACS.

Due to the limited osteogenic capacity of bone marrow mesenchymal stem cells (BMSCs) in elderly patients, treating elderly patients in need of bone repair has always been a challenging medical problem. Here, Wang et al. performed bone repair by means of the mechanical action of body−driven wearable triboelectric nanogenerators (WP-TENG) and piezoelectric ceramics (Figure 9a) [71]. To improve the friction performance of the equipment, nylon sheets were selected as the base material; soft foam was attached to the nylon to increase the friction, polytetrafluoroethylene (PTFE) film and aluminum foil were used as positive and negative friction layers, and two copper foils were attached to the back of the two friction layers as electrodes. The peak current reached 30 µA, and a good recovery effect on elderly bone marrow mesenchymal stem cells was demonstrated. WP−TENGs can also be customized, and can be produced in different shapes and sizes to meet the personalized needs of elderly patients, improving their quality of life when undergoing long-term treatment.

With advances in society and science, alopecia has become an increasingly common modern disease [72,73]. Unlike acute illnesses that can take effect quickly in a short period of time, alopecia often requires long-term treatment. Here, Yao et al. designed a wearable electrical stimulation device (M-ESD) activated by body movement [74] to promote hair regeneration. Its working principle is the improvement of the secretion of growth factors in blood vessels through electrical stimulation (Figure 9b), thereby relieving hair keratin disorder and increasing the number of hair follicles. The M-ESD consisted of two modules: a TENG with an electric pulse generator function and a pair of interlaced electrodes. This TENG had two friction layers that were connected by a soft Ecoflex belt. Because Ecoflex allows arbitrary tension, bending, and torsion within the ~900% strain limit, the TENG was able to perform omnidirectional energy collection as a result of the adaptive design described above.

#### 4.1.3. Muscle Tissue Function Repair

Loss of muscle function can lead to many diseases, including stroke, spinal cord injury, and multiple sclerosis [75,76]. Electrical stimulation has positive effects when treating this disease. However, the long treatment cycles can be inconvenient for patients. To coordinate the relationship between the treatment cycle and quality of life, flexible and stable biomedical equipment that is able to meet the individual needs of patients is essential. Wang et al. developed a neural stimulation system that used stacked TENGs as the power supply (Figure 9c) [77]. This system had the characteristics of small size, customizability, etc., and could easily be applied for long-term treatment. The TENGs were made of PET sheets folded into a zigzag structure. This special structure stored energy in the form of elastic properties, and the surface of each PET sheet was attached to an aluminum film as an electrode. At the same time, to improve the efficiency of power generation and meet the electrical demand over a long course of treatment, polytetrafluoroethylene film (PTFE) with high electronegativity was attached to the top of some of the aluminum films as a friction layer. The system used a new flexible multi-channel intramuscular electrode as a universal neural interface. Efficient muscle stimulation was successfully achieved using the TENGs. On this basis, it is expected that this system could be used in cases of loss of muscle function to help patients recover their ability to exercise.

### 4.2. Organ Treatment

Organs include not only the heart, liver, and lungs, but also the outermost organ of the human body: the skin. At present, the treatment of minor internal organs and skin injuries is heavily dependent on the patient’s self-healing, with severe cases requiring surgical treatment, ligation, hemostasis, and drug control. However, the rise of electric stimulation therapy presents another option for organ repair. At the same time, biomedical equipment based on TENGs are characterized by their flexibility and self-powered nature, can provide effective treatment over long periods of time, and can be customized to a certain extent according to a patient’s individual circumstances, thus providing an effective route for personalized treatment of organs.

#### 4.2.1. Cardiac Treatment

**Figure 10:**
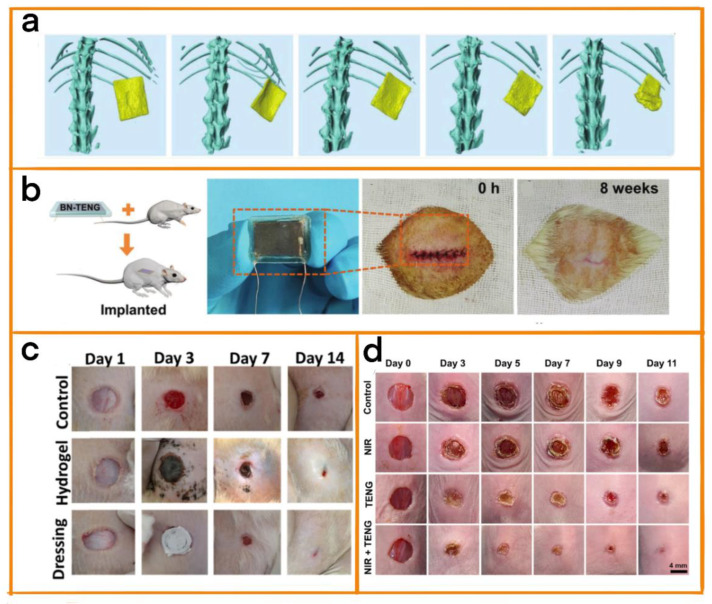
(**a**) Biodegradation in rats. (**b**) BN-TENG implantation: pictures of BN-TENG and the change in state at the site of implantation after suture. Reprinted with permission from [81]. 2018, John Wiley and Sons. (**c**) Representative images of wounds on days 1, 3, 7, and 14. Reprinted with permission from [82]. 2022, Elsevier. (**d**) Images of skin wounds from different treatment groups taken on days 0, 3, 5, 7, 9, and 11. Reprinted with permission from [83]. 2022, Elsevier.

The heart is the center of the blood circulatory system, and plays a vital role in our body. In addition, compared with conventional acute diseases, it often takes longer for initial results to be achieved in heart−related diseases, resulting in the treatment process having higher biosafety requirements [78,79,80]. Therefore, Jiang et al. developed triboelectric nanogenerators (BN TENGs) that can be fully bio-absorbed for the treatment of bradycardia and arrhythmia using natural materials [81]. The friction layer of the equipment was made of cellulose, chitin, rice paper (RP), silk fibroin (SF), and egg white (EW), and ultra−thin Mg film was used as the electrode. To improve the power generation efficiency, the surface of the friction layer was treated using inductively coupled plasma reactive ion etching (ICP), thus increasing the contact area. Finally, SF film was used as the encapsulation layer. Importantly, this material has been proven to be degradable, and produced no obvious infection symptoms after implantation into rats (Figure 10b). Due to the use of natural materials and the high plasticity of the device, it is able to meet the individual needs of patients, and it thoroughly degrades and is absorbed by the body after the completion of its functions, thus improving biological safety (Figure 10a).

#### 4.2.2. Skin or Wound Healing

As the outermost organ of the human body, the skin is much more likely to be injured than other organs. Wound healing is a long-term process, and the position and size of the wound is fraught with uncontrollability. It is therefore necessary that the corresponding healing equipment be able to be operated over long periods of time and be customizable in order to meet the different needs of different patients. Therefore, Sharma et al. proposed an interactive wound dressing based on TENGs [82] that provided an electrical stimulation environment for patients, accelerating wound healing (Figure 10c). The dressing consisted of carbonized polydopamine/polydopamine/polyacrylamide and was paired with polyvinylidene fluoride (PVDF) film. Therefore, the dressing exhibited mechanical strength and plasticity similar to that of muscle, and could be used in various scenarios. Since the hydrogel also provides a moist and effective wound environment, the TENG can help repair wounds that have difficulty healing under the effects of electrical stimulation, such as diabetic foot ulcers.

Du et al. designed a single-electrode working mode skin patch [83] that used electrical stimulation and photothermal heating to promote wound healing (Figure 10d). Polypyrrole/Pluronic F127 hydrogel was used as an electrolyte. In such hydrogel electrolyte designs, PPy endows the electrolyte with good conductivity and excellent photothermal conversion performance, while F127 endows the electrolyte with low modulus, continuity, and shape adaptability. In addition, the process of treatment using the skin patch could also be combined with photothermal heating technology to provide a suitable temperature for wound healing.

### 4.3. Bacterial Infection

#### 4.3.1. Long-Term Bacterial Eradication

**Figure 11:**
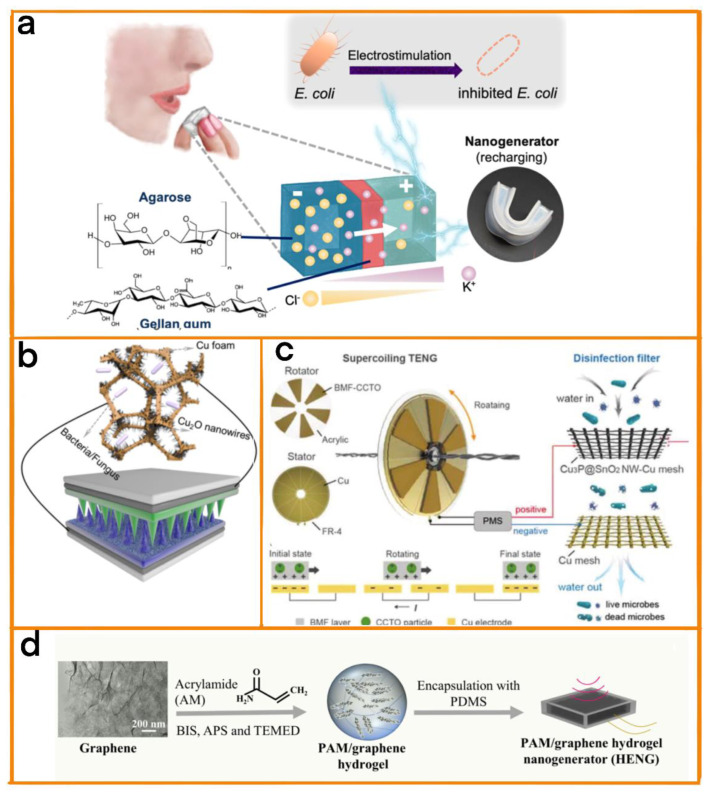
(**a**) Schematic diagram of a cube polysaccharide battery composed of the soft natural hydrogel. Reprinted with permission from [84]. 2021, Elsevier. (**b**) Schematic diagram of sterilization system. Cu_2_O and Staphylococcus aureus. Reprinted with permission from [85]. 2022, Elsevier. (**c**) Schematic diagram of S-TENG disinfection system, including an S-TENG, a power management system (PMS) with rectifier, and a disinfection filter for inactivating microorganisms in water. Reprinted with permission from [86]. 2022, John Wiley and Sons. (**d**) The schematic diagram of ultrasonic-driven vagus nerve electrical stimulation based on implanted soft HENG and the manufacturing process of HENG. Reprinted with permission from [90]. 2022, Elsevier.

The continuous and effective elimination of bacteria over longer periods of time is one of the main ways of fighting bacterial infection. Thanks to its flexibility and self-powered nature, TENG-based sterilization equipment can be customized for placement in areas with a high incidence of bacterial infection in order to block pathways of bacterial infection, and can be used to maintain sterile conditions in humans through the selection of materials with good biocompatibility. In this context, Lin et al. developed an edible battery with good biocompatibility (Figure 11a) [84]. The battery was made of a hydrogel and was powered using a self-charging TENG. The TENG consisted of a tooth-like polyethylene terephthalate (PET), and the PET was uniformly covered with a Pt film. In addition, to increase its biocompatibility, the top of the Pt was also covered in PTFE and a chitosan/glycerol film. The device generated a maximum voltage of 300 mV and was able to kill or eliminate 90% of bacteria (including Escherichia coli) within 30 min. Therefore, the battery could be used to fight against drug−resistant bacteria in the stomach and intestines. This battery shows great potential, and can be easily applied in the long-term treatment of gastrointestinal bacterial infection.

Feng et al. prepared a TENG with high power output that was able to achieve a sterilization efficiency of 98% [85]. The friction layer of the TENG consisted of a fluorinated polyurethane (F-PU) layer with a surface micronucleus prepared using maskless direct image lithography (DIL) technology and trichlorosilane (FOTS) steam (Figure 11b). Compared with TENGs with a flat structure, the developed TENG achieved an increase in output power of 400%, and the sterilization efficiency obtained was 98%.

Huo et al. also developed a new disinfection system for bacteria and viruses [86]. The disinfection system consisted of a supercoiling−mediated rotational triboelectric nanogenerator (S-TENG), a power management system with a rectifier, and a disinfection filter for the inactivation of microorganisms in water. In order to improve the efficiency of power generation, the S-TENG was constructed using six butyl melamine formaldehyde (CCTO-BMF) friction layers doped with CaCu_3_Ti_4_O_12_ particles, and was able to achieve an ultra-fast speed of 7500 rpm for driving a new oxidation-assisted electroporation mechanism (Figure 11c). Thanks to its ultra−high rotation speed, it was able to simultaneously realize a nanowire-enhanced local electric field and the generation of oxidative species, inactivating 99.9999% of microorganisms at a high flux of 15,000 L h^−1^ m^−2^. This rapid, efficient and stable method for the long-term disinfection and sterilization of viruses meets the requirements of emergency water disinfection and the sterilization of portable medical equipment in areas with power shortages.

#### 4.3.2. Anti-Inflammatory Treatment of Sepsis

Bacterial infection is one of the essential sources of disease [87,88,89]. With the abuse of antibiotics and the emergence of various drug-resistant bacteria, the development of new targeted treatment methods is an urgent need. In this context, Chen et al. designed a vagus nerve stimulator based on an implantable high-performance hydrogel nanogenerator (HENG) [90]. The nanogenerator was based on a polyacrylamide/graphene conductive hydrogel, and generates alternating current through ultrasonic-induced vibration at the conductive hydrogel/electrolyte interface. Without the use of an auxiliary rectifier, the subcutaneous implanted HENG can be used directly as a wireless nerve stimulator, and the current density and waveform can be programmed with the use of external ultrasonic pulses to inhibit the release of pro-inflammatory cytokines for the long-term anti−inflammatory treatment of sepsis (Figure 11d).

### 4.4. Long-Term Auxiliary Physical Therapy

#### 4.4.1. Adjuvant Treatment of Hearing Impairment

**Figure 12:**
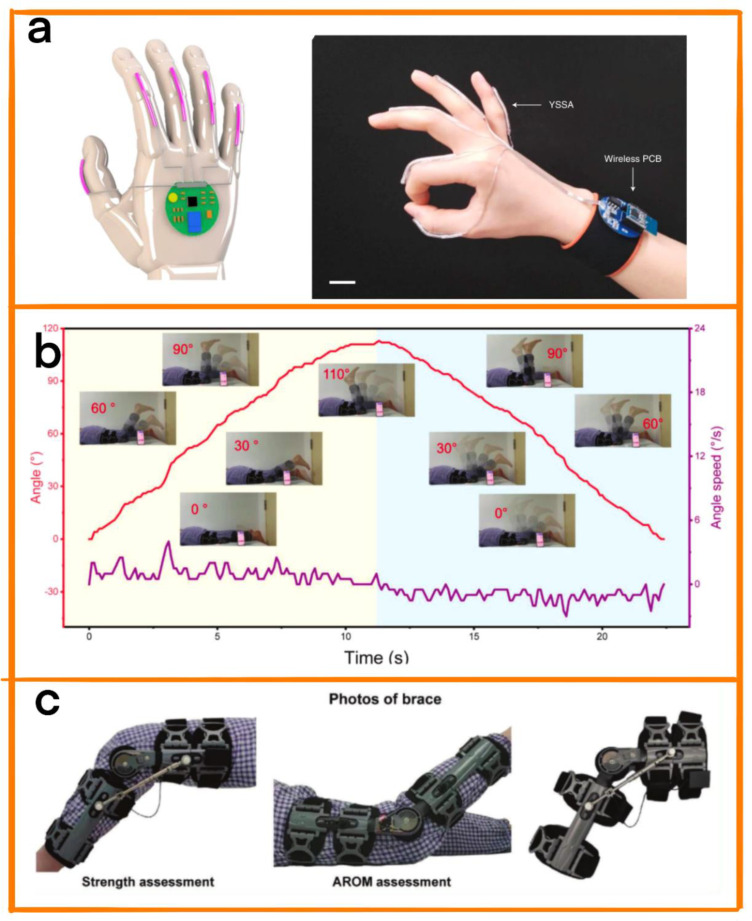
(**a**) Schematic diagram of a wearable sign language voice translation system. Reprinted with permission from [91]. 2020, Elsevier. (**b**) Real−time angle signal, angle speed, and photos within the range of motion. (**c**) Schematic diagram of rehabilitation brace system. Reprinted with permission from [93]. 2022, Elsevier.

With the development of TENGs, physical auxiliary treatment of hearing impairment has also taken a new direction. Thanks to the flexibility and self-powering characteristics of TENGs, physical auxiliary equipment based on TENGs can be customized in terms of shape and materials depending on the personal needs of patients. Zhou et al. designed a TENG-based symbol-to-speech translation glove to help people with language disabilities carry out long-term physical therapy [91]. The sensing unit of the glove was composed of conductive yarn wound on the rubber fiber. A plurality of sensing units formed a scalable sensor array (YSSA) (Figure 12a). Due to its helical structure, the TENG is able to meet the individual needs of different patients, and can be woven into different shapes. At the same time, by means of a machine learning algorithm, the sign language recognition rate of the YSSA was improved to 98.63%, and it was possible to achieve real-time conversion of sign language into language. Yuce et al. designed an eye movement sensor based on TENGs for the long-term physical auxiliary treatment of paralyzed people [92]. The TENG, which was integrated into the human–computer interface, detected eyelash movement by identifying the direct triboelectric interaction between hair and silicone to help paralyzed people communicate. In addition, due to the plasticity of the raw materials, the device is also able to meet the personalized needs of different patients through designs using different shapes, thus improving the long-term wearing comfort for patients.

#### 4.4.2. Adjuvant Treatment of Knee Osteoarthritis

Due to the increasing aging of the world’s population, diseases of the elderly are becoming more common. Knee osteoarthritis is one of the most common diseases among the elderly, and can be treated using total knee arthroplasty (TKA). To improve the patient’s quality of life after surgery, Luo et al. proposed a portable self-evaluation stent for the rehabilitation of TKA patients (Figure 12c) [93]. The friction layer of the TENG consists of Kapton film, with a thickness of about 50 μm. In addition, the whole TENG sensor was produced using Printed Circuit Board (PCB) technology, making it suitable for use in prolonged auxiliary physical therapy. The system can be used to detect the knee bending angle and isometric muscle strength of patients over a long period of time, and to conduct targeted treatment of patients on the basis of data analysis (Figure 12b).

### 4.5. Drug Delivery

**Figure 13:**
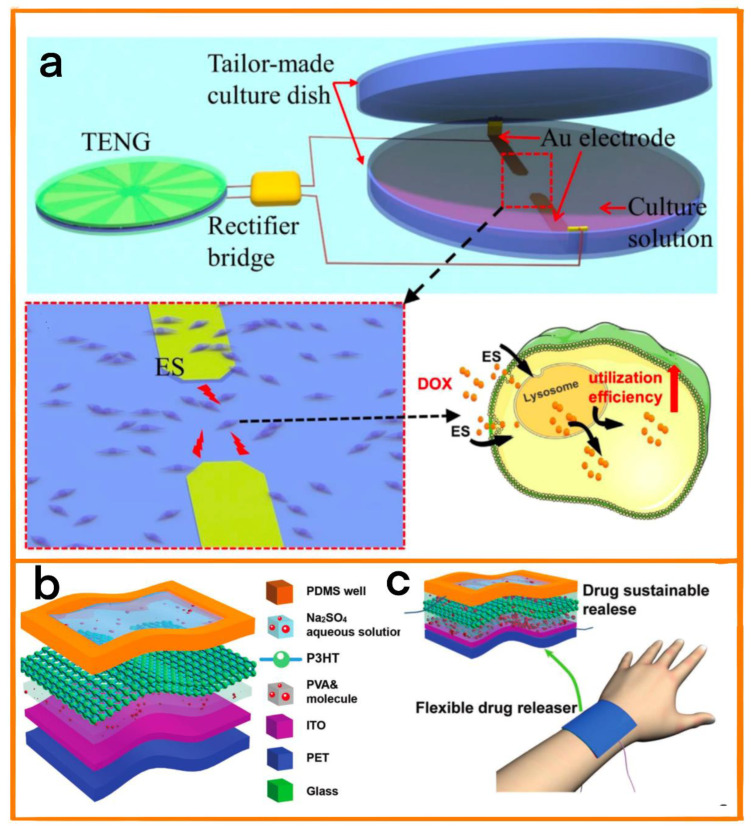
(**a**) Diagram of the equipment and schematic diagram of the co-culture of cells and DOX after electrical stimulation. Reprinted with permission from [94]. 2022, Frontiers. (**b**) Structural diagram of this TENG. (**c**) Schematic diagram of the sustainable release of salicylic acid. Reprinted with permission from [95]. 2020, John Wiley and Sons.

When treating long-term diseases such as cancer, an accurate drug delivery system is essential to reduce side effects and improve treatment efficiency. To this end, Chen et al. designed a self-powered disc-shaped TENG (D-TENG) [94]. The disc-shaped TENG used PVDF and PA as friction layers, and Cr/Ag electrodes were attached to the bottom of the PVDF. As a result of its special disc-shaped design, it was able to obtain energy from the surrounding environment through rotation, and contained a pair of gold electrodes that could be used to electrically stimulate cells in the electrodes. Upon electrical stimulation of cancer cells by the drug delivery system, the chemotherapy drug doxorubicin (DOX) was significantly absorbed by the cancer cells (Figure 13a). Therefore, the use of new TENG devices for the precise treatment of some long-term diseases shows broad prospects.

**Table 3:** Summar of TENG treatment parameters.

Date	Positions	Sizes [cm^2^]	Materials	Energy Sources	Outputs	Applications	Working Modes
2022 [69]	Sciatic nerve, rat	3.5 × 2.5	PDMS, PA6	Respiratory drive	0.13 μA	Nerve repair	Contact-separation
2021 [70]	Finger	0.5 × 0.5	PMDS, Ny, CAB	Tactile pressure	2.5 V	Tactile recovery	Contact-separation
2022 [71]	Bone	None	PTFE, Al	Movement	30 µA	Bone repair	Lateral sliding
2019 [74]	Head, rat	2 × 2	PET, PTFE	Movement	430 mV	Hair regeneration	Lateral sliding
2019 [77]	Muscle	10 × 10	PTFE, PET	Movement	95 μW	Muscle repair	Contact-separation
2018 [81]	Heart	1 × 2	Celllose, Chitin, RP, SF, and EW	None	0.6 µA	Heart disease treatment	Contact-separation
2022 [82]	Wound	None	PDA, PVDF	Press	42 mV	Wound repair	Contact-separation
2022 [83]	Wound	4 × 4	PPy	Press	33 mW m^−2^	Wound repair	Contact-separation
2021 [84]	Stomach	10	PET, PTFE, Pt	Micro vibration	185 mV	Sterilization	Contact-separation
2022 [85]	None	5 × 5	Fluornated Polyurethane	Press	22 μA	Sterilization	Contact-separation
2022 [86]	Solution	None	CCTO-BMF, Cu, Acrylic	Gravity	322 µA	Sterilization	Contact-separation
2021 [90]	Subcutaneous, rat	1.5 × 1.5	Polyacrylamide, Graphene	Ultrasonic drive	1.6 mA	Anti inflammatory Treatment of sepsis	Contact-separation
2020 [91]	Hand	None	Polyester, PDMS	Hand movement	2.47 V	Auxiliary physical Therapy	Contact-separation
2022 [93]	Knee	None	Kapton, Cu	Movement	35 V	Treatment of knee osteoarthritis	Contact-separation
2022 [94]	Internal environment	None	PVDF, PA Cr/Ag electrode	Micro vibration	3.7 μA	Promote drug absorption	Lateral sliding
2020 [95]	Internal environment	None	PDMS, PET, PVA	Micro vibration	165.6 μA	Drug transportation	Contact-separation

Liu et al. also designed a self-powered drug delivery device (FDRD) [95]. The device integrated three TENG units, each consisting of multi-layer flexible structures (Figure 13b) that effectively collect disordered biological energy from the environment, providing electrical power for the device. At the same time, the device monitors the flow of small molecules (such as salicylic acid, which has exfoliating, bactericidal, and anti-inflammatory effects) out of the FDRD in real time (Figure 13c), and provides vital data support for the long-term personalized treatment of patients by recording and analyzing the flow rate of small molecules.

## 5. Conclusions and Perspectives

In the current paper, recent advances in TENG-based medical equipment for the diagnosis and long-term treatment of disease have been concisely introduced, and the corresponding design strategies, including design concepts, operation principles, and the problems and solutions of the designs, have been described in detail. For better understanding, the developed self-powered multifunctional systems described under the categories of bioenergy collection, real-time diagnosis, and long-term treatment. In addition to power enhancement measures, TENGs have also been developed with flexible structures so that they can be integrated with other electronics to achieve a perfect fit to skin or can be implanted. Meanwhile, TENGs can also be constructed using different raw materials to improve their biological safety and antibacterial performance and reduce discomfort to the body.

However, currently, having reached a stage beyond exploratory research, there are huge obstacles to achieving practical application of TENG-based clinical systems that persist due to the well-defined test environments in labs. Some of these shortcomings may arise from the systems, while some shortcomings lie in TENGs themselves.

### 5.1. TENG Energy Collection

#### 5.1.1. Increased Output Power

When considering clinical applications, there are three main ways of improving the power output of devices: modifying the surface of the friction layer, choosing a biocompatible friction layer with a high surface charge, and changing the overall structure of the TENG.

Techniques for achieving the surface modification of the friction layer can be categorized into chemical and physical methods, whereby chemical processes involve the grafting of chemical groups to assist in electron transmission, while physical methods primarily employ modeling, laser engraving, plasma processing, etc., to change the surface contact area of the friction layer. The correct combination of the two approaches can be used to improve the output power.

For the long-term power supply of medical devices, in the future, it will be necessary to focus on finding materials with higher friction electronegativity and pay attention to the biological safety of new materials. When modifying the surface of the friction layer, it is necessary to find surface structures that can be adapted to the human body’s internal environment in order to reduce rejection. The overall structure of TENGs is also an essential factor affecting triboelectricity and biocompatibility. Additionally, the design of structures that can achieve greater integration with the human body, that are more comfortable to wear, and that are more in line with physical laws is also a major future research direction.

#### 5.1.2. Stability

It is essential to maintain a stable power supply in the treatment of long-term diseases. Most TENGs generate electricity through continuous contact between layers. Therefore, the corresponding loss in the friction layer is significant. Therefore, in order to reduce this loss and improve the service life and stability of TENGs, attention should be paid in the future to the identification of wear-resistant materials or new friction methods with low rates of loss.

In addition, some TENGs and medical devices need to be integrated into the human internal environment. Therefore, the need to improve the service life of TENGs in the human internal environment should be taken into account. Thus, in the future, acid- and alkali-resistant materials should be employed, or existing materials should be modified in order to improve their acid and alkali resistance. Secondly, waterproof coatings could also be added to the materials in order to reduce the impact of humid environments on the efficiency of power generation using TENGs. When using TENGs for medical diagnosis, various complex minor disturbances in the human body can seriously affect the stability of the devices. Therefore, in the future, interference could be reduced or eliminated by adding filter circuits.

### 5.2. TENGs for Diagnosis

#### 5.2.1. Wireless Data Transmission

For long-term monitoring of diseases, it is necessary to continuously collect relevant data for further analysis. Currently, due to the relatively bulky nature of traditional cables, wireless transmission is mostly used. However, wireless communication is highly dependent on the chip, and its energy consumption is relatively high, which affects the stability of the device. Therefore, a future research direction would be a focus on the selection of more energy-efficient wireless transmission methods, such as by using the electrostatic induction effect to drive nearby coils via TENGs to achieve signal transmission. However, due to the significant internal resistance of TENGs, the transmission effect of this technology is not ideal. Therefore, in the future, the internal resistance of TENGs could be improved, making it possible to use TENG drive coils for signal transmission.

#### 5.2.2. Higher Sensitivity

With the continuous development of modern medicine, greater sensitivity is required for the diagnosis of some diseases. To improve the sensitivity of devices in the future, more sensitive materials can be developed, and interference from the environment can be reduced by employing multiple groups of filter circuits. At the same time, accurate structural customization is also an important method of reducing errors in the systems. For example, overall structural error can be reduced through the use of high-precision 3D printing or laser etching.

#### 5.2.3. Comfortability

Since diagnostic equipment needs to be worn for a long time, its comfort is of great importance. In this regard, new methods and materials should be explored to improve the comfort of the equipment. For example, the use of improved raw materials such as natural fibers such as cotton, silk, hemp, and cotton silk can achieve properties such as air permeability and hydrophobicity. It is widely known that a high degree of customizability will lead to an enhanced degree of fitting between the device and the skin. Similarly, from the perspective of overall design and aesthetics, personalized design of the appearance can be carried out in combination with clinical experiments to enhance practicability.

### 5.3. TENG for Treatment

#### 5.3.1. Biosafety

Output power is not the only indicator of TENG in long-term treatment. Biosafety is very important for improving treatment quality for patients and reducing pain and side effects. Currently, the mainstream option is for biocompatible encapsulation materials in order to avoid human contact with the TENG equipment. However, due to the complex structure of the human body, encapsulation materials cannot guarantee that leakage will not occur, especially in the case of implantable treatment equipment. Human bodily fluids can flow into TENG equipment, affecting its normal functioning, while also resulting in a human rejection reaction. Therefore, it is important to carefully select the raw materials and optimize the structure of the packaging layer to improve environmental tolerance. In addition, assessment of long-term biosafety should be carried out with respect to manufacturing methods and raw materials in order to reduce the occurrence of accidents.

#### 5.3.2. Multifunctionality

For medical devices that need to be worn for a long time, increasing the number of functionalities of TENGs is of great significance for improving patients’ quality of life. For example, the integration of power supply, sensing, treatment, and other functions could be considered in these devices. This could lead to optimization of the volume of the equipment and reduce unnecessary time wastage, as well as being more convenient and straightforward. However, due to the requirements of convenience, space in such equipment is relatively limited, so seamlessly integrating these functions in practical application remains a challenge. Secondly, with the increasing integration of processes, such treatment equipment will require the supply of larger amounts of power, and it will also be necessary to improve the output power of TENGs. By achieving miniaturization, integrated multifunctional treatment devices would undoubtedly be of great help in the long-term treatment of disease.

#### 5.3.3. Degradability

One of the advantages of degradable technology is that device removal surgery, which may lead to secondary infection, does not need to be carried out. Therefore, the risk of infection due to long-term treatment with implantable devices can be significantly reduced. Still, due to the complexity of the human environment, the output performance and degradability of TENGs need to be carefully balanced, and blindly pursuing degradability may result in a decrease in the service life of the TENG. Based on the current research, there are a number of natural materials that are good options, including cellulose, chitin, etc., as they can be absorbed by the human body, thus reducing unnecessary side effects. However, systematic research is still needed regarding their chemical properties, and finding the optimal balance between degradation time and output performance is still a big challenge.

## Figures and Tables

**Table 1:** Summary of parameters of wearable and implantable TENGs.

Date	Positions	Sizes [cm^2^]	Materials	Energy Sources	Outputs	Applications	Working Modes
2022 [41]	Wearable	None	Cellulose particles	Particle vibration	70 μW	Electricity generation	Contact-separation
2019 [42]	Foot	2 × 2	Al	Walk	1.67 μW	Electricity generation	Contact-separation
2022 [43]	Nose	None	CNT, Wire	Breathing	150 V	Respiratory monitoring	Contact-separation
2021 [44]	Wearable	4 × 4	Sponge, PANI	Vibration	280 μW	Electricity generation	Contact-separation
2021 [49]	Heart, rat	1.2 × 1.2	Ecoflex	Heart beating	51.74 nA	Biomedical monitoring	Contact-separation
2018 [50]	Stomach, rat	1 × 2	PDMS, PI	Stomach Peristalsis	40 μW	Nerve stimulation	Contact-separation
2019 [51]	Muscle, rabbit	1.5 × 2	Titanium	Muscle vibration	80 nA	Biomedical monitoring	Contact-separation
2018 [52]	Heart, pig	1.0 × 1.5	Al, PTFE PDMS	Heart beating	6.2 V	EP monitoring	Contact-separation

## Data Availability

Data available on request from the authors.
